# Analysis of human prostate cancers and cell lines for mutations in the TP53 and KLF6 tumour suppressor genes

**DOI:** 10.1038/sj.bjc.6601164

**Published:** 2003-08-12

**Authors:** K-R Mühlbauer, H-J Gröne, T Ernst, E Gröne, R Tschada, M Hergenhahn, M Hollstein

**Affiliations:** 1 Department of Genetic Alterations in Carcinogenesis, German Cancer Research Center (Deutsches Krebsforschungszentrum), D-69120 Heidelberg, Germany; 2 Department of Cellular and Molecular Pathology, German Cancer Research Center (Deutsches Krebsforschungszentrum), D-69120 Heidelberg, Germany; 3 Urology Clinic, Diakonissenkrankenhaus, D-68163 Mannheim, Germany

**Keywords:** suppressor genes, point mutations, prostate tumours

## Abstract

A recent report suggests that the KLF6 gene encoding the Krüppel-like factor 6 protein is a frequently mutated, putative tumour suppressor gene in prostate cancer. The aims of the present study were to confirm these initial findings by determining the frequency of exon2 KLF6 mutations in a cohort of European prostate cancer patients, and to investigate whether there was evidence for mutational inactivation of both the KLF6 and TP53 tumour suppressor loci in some tumours. We examined 32 primary prostate tumours and three prostate tumour cell lines for mutations by PCR amplification and direct dideoxy sequencing (KLF6), and by oligonucleotide microarray (p53GeneChip™) analysis and dideoxy sequencing (TP53). Whereas TP53 mutations typical of prostate cancer were found at a frequency consistent with the literature, no KLF6 mutations were found in any of the tumour samples nor in the three prostate cancer cell lines.

Krüppel-like factors serve as core transcription factors and participate in regulation of numerous mammalian genes (Bieker, 2001; Black *et al*, 2001). One of the factors, KLF6, is thought to play a role in repair of vascular injury and in tissue remodelling (Kojima *et al*, 2000). Very recent findings have suggested that the KLF6 protein also shares several intriguing features in common with the TP53 tumour suppressor protein. Their tumour suppressor properties rely at least in part on the ability to regulate transcription of growth control genes, and loss of function in neoplastic development typically occurs when a missense mutation arises in one allele, and the remaining allele is lost (Hainaut and Hollstein, 2000; Vogelstein *et al*, 2000; Narla *et al*, 2001). In addition, the two proteins have in common at least one important transcriptional target, WAF1/p21, an inhibitor of cyclin-dependent kinases and regulator of cellular growth arrest. In prostate cancer, inactivating TP53 mutations are detected at frequencies in the range of 10–20% in primary tumours (IARC TP53 Mutation Database: www.iarc.fr/P53/index.html1), whereas based on the novel findings of Narla *et al* (2001), tumour-specific KLF6 mutations would be expected overall in up to half of sporadic prostate tumours with Gleason scores in the range of 3–8 (not preselected for loss of heterozygosity status), and thus would constitute the most frequent gene mutation event identified to date in prostate carcinogenesis. If mutations that compromise function of the KLF6 transcription factor are indeed common in prostate cancer, we might then ask whether clustering analysis of data from molecular profiling of tumours (Ernst *et al*, 2002) would define KLF6 mutant tumours as a subgroup.

## METHODS

We examined tumour material from 32 patients from Germany, most of whom had disease with a Gleason score of 7 or higher (Table 1

**Table 1:** KLF6 and TP53 gene mutation analysis of prostate tumours from European patients

						**Mutation analysis results**		
**Patient**	**Age (years)**	**Tumour classification**	**Grading**	**Gleason score**	**Tumour cell recovery**	**KLF6 (ddSeq)**	**TP53 (Array)**	**TP53 (ddSeq)**
#1	78	pT3a, pN0, pMx	G2	7	Microdissection	Neg.	Neg.	
#2	78	(not classifiable)	G3	9	Microdissection	Neg.	c.190 CCT/CTT	c.190 CCT/CTT
#3	64	pT2b, pNx, pMx	G2	6	Microdissection	Neg.	ND	
#4	73	pT3b, pN1, pMx	G3	9	Microdissection	Neg.	ND	
#5	83	pT1b, pN0, pMx	G2–G3	7	Microdissection	Neg.	Neg.	
#6	73	pT3a, pN0, pMx	G2–G3	8	Microdissection	Neg.	Neg.	
#7	65	pT2b, pN0, pMx	G1–G2	5	Microdissection	Neg.	Neg.	
#8	70	pT3a, pN0, pMx	G2	6	Microdissection	Neg.	Neg.	
#9	73	pT2, pN0, pMx	G2–G3	8	Microdissection	Neg.	Neg.	
#10	72	pT2a, pN0, pMx	G2	7	Microdissection	Neg.	Neg.	
#11	63	pT2b, pNx, pMx	G2	7	Laser capture	Neg.	ND	
#12	77	pT3a, pN0, pMx	G2	7	Laser capture	Neg.	ND	
#13	64	pT4, pN0, pMx	G3	9	Laser capture	Neg.	ND	
#14	71	pT3b, pN0, pMx	G2	7	Laser capture	Neg.	Neg.	
#15	64	pT3b, pN1, pMx	G3	9	Laser capture	Neg.	ND	
#16	62	pT3, pN0, pMx	G2	7	Laser capture	Neg.	ND	
#17	71	pT2b, pN0, pMx	G2–G3	7	Microdissection	Neg.	Neg.	
#18	75	pT2a, pN0, pMx	G1	4	Microdissection	Neg.	c.285 GAG/AAG	c.285 GAG/AAG
#19	65	pT3a, pN0, pMx	G2–G3	7	Microdissection	Neg.	Neg.	
#20	71	pT3b, pN0, pMx	G2–G3	7	Microdissection	Neg.	Neg.	
#21	61	pT3a, pN0, pMx	G2–G3	6	Microdissection	Neg.	Neg.	
#22	73	pT2b, pN0, pMx	G2–G3	8	Microdissection	Neg.	Neg.	
#23	64	pT3a, pN0, pMx	G2–G3	8	Microdissection	Neg.	Neg.	
#24	72	pT3b, pN0, pMx	G1–G3	8	Microdissection	Neg.	Neg.	
#25	70	pT3a, pN0, pMx	G2	6	Microdissection	Neg.	Neg.	
#26	66	pT3b, pN0, pMx	G2–G3	8	Microdissection	Neg.	Neg.	
#27	59	pT2b, pN0, pMx	G2–G3	8	Microdissection	Neg.	Neg.	
#28	66	pT2b, pN0, pMx	G2	7	Microdissection	Neg.	Neg.	
#29	67	pT3a, pN1, pMx	G2–G3	8	Microdissection	Neg.	c.282 CGG/TGG	c.282 CGG/TGG
#30	66	pT3b, pN1, pMx	G2–G3	8	Microdissection	Neg.	Neg.	
#31	66	pT3b, pN1, pMx	G2–G3	8	Microdissection	Neg.	Neg.	
#32	64	pT3b, pN1, pMx	G3	9	Microdissection	Neg.	Neg.	

ddSeq=Sanger dideoxy sequencing; Array=Affymetrix P53 GeneChip; Neg.=negative; ND=not done.

). Tumour areas with >90% neoplastic cellularity were either microdissected from 5 *μ*m tissue sections cut from buffered formalin-fixed paraffin-embedded material, or tumour cells were catapulted from sections of snap-frozen tumour tissue with a P.A.L.M. laser device (P.A.L.M. Microlaser Technologies, Bernried, Germany) as indicated in Table 1. To avoid the hazards of polymerase chain reaction (PCR) contamination, genomic DNA was extracted in a laboratory designed for that purpose in which neither PCR reactions are performed nor PCR products handled. Polymerase chain reaction set-up was performed in a second laboratory in a special hood equipped with UV lamps that were illuminated for 30 min before the beginning of each PCR experiment. Exon 2 of the KLF6 gene (the exon with the longest coding sequence and in which all but two KLF6 mutations were reported previously) was amplified with primers we designed to optimise amplification (KLF6Ex2F and KLF6Ex2R, Table 2

**Table 2:** Primers used in this study

**Name**	**Gene**	**Sequence**
KLF6Ex2F	KLF6	5′ CGGGCAGCAATGTTATCTGTCCTTC 3′
KLF6Ex2FA	KLF6	5′ TCGTCATGGCAATCACGTGCCTTC 3′
KLF6Ex2R	KLF6	5′ CGGCTCCCTCCAGGGCTGGTGCA 3′
KLF6Ex2FC	KLF6	5′ CCCACGGCCAAGTTTACCTCCGACC 3′
KLF6Ex2RC	KLF6	5′ GGAGCTCAATTTTCCCGAGCTGACC 3′
GCEx8F	TP53	5′ GTAGGACCTGATTTCCTTACTGCCTCTTGC 3′
GCEx8R	TP53	5′ ATAACTGCACCCTTGGTCTCCTCCACCGC 3′
GCEx5F	TP53	5′ CTTGTGCCCTGACTTTCAACTCTGTCTC 3′
GCEx5R	TP53	5′ TGGGCAACCAGCCCTGTCGTCTCTCCA 3′
GCEx6F	TP53	5′ CCAGGCCTCTGATTCCTCACTGATTGCTC 3′
GCEx6R	TP53	5′ GCCACTGACAACCACCCTTAACCCCTC 3′

Multiplex PCR for P53GeneChip analysis was performed with the P53 Primer Set from Affymetrix.

). Reactions were performed in 1 × PCR buffer from Boehringer Mannheim (final concentration of MgCl_2_: 1.5 mM), 200 *μ*M dNTPs, and 2.5 U *Taq* polymerase (Boehringer Mannheim, Germany) in a total volume of 50 *μ*l. Amplification was performed in an MJB thermal cycler programmed for 40 cycles (95°C, 1 min; 60°C, 1 min; 72°C 1 min), and included an initial denaturation step at 95°C for 2 min, and a final elongation step of 5 min at 72°C. Polymerase chain reaction products were purified with Microcon 100 filters from Millipore (Eschborn, Germany) and used as template in dideoxy cycle sequencing reactions with fluorescent dye-labelled dideoxynucleotides and DNA polymerase from Applied Biosystems International (ABI, Weiterstadt, Germany). Four cycle sequencing reactions were performed, using primers KLF6Ex2FC, KLF6Ex2RC, KLF6Ex2R and KLF6Ex2FA (Table 2), which allowed accurate reading of both DNA strands of all of exon 2 (571 bp) and adjacent splice sites. For 28 of the 32 tumours, there was sufficient material to allow two independent amplification reactions of exon 2 from genomic DNA, and sequencing of the entire fragment in both directions. Capillary electrophoresis of sequencing reaction products was performed with the ABI Model 310 Genetic Analyzer.

TP53 mutation analysis was performed on tumours from 25 of the patients, using aliquots of the same genomic DNA samples that were tested for KLF6 mutations, but employing reagents for multiplex PCR of the TP53 gene. Reagents and p53GeneChips were purchased from Affymetrix (Santa Clara, CA, USA) and used according to recommendations from the manufacturer. Chips were scanned with a Hewlett-Packard GeneArray scanner and signal intensities were evaluated with Affymetrix GeneChip software. The probe array methodology scans all coding exons and splice sites of the human p53 gene for point mutations with high efficiency and accuracy (Ahrendt *et al*, 1999; Wen *et al*, 2000). To confirm results, tumours screened with the p53GeneChip that yielded mutant signals were sequenced by standard procedures similar to those used above for KLF6, that is, PCR amplification of individual exons from genomic DNA using primers listed in Table 2, and dideoxy cycle sequencing, as we have described previously for the TP53 gene (Biramijamal *et al*, 2001).

## RESULTS AND DISCUSSION

**Figure 1:**
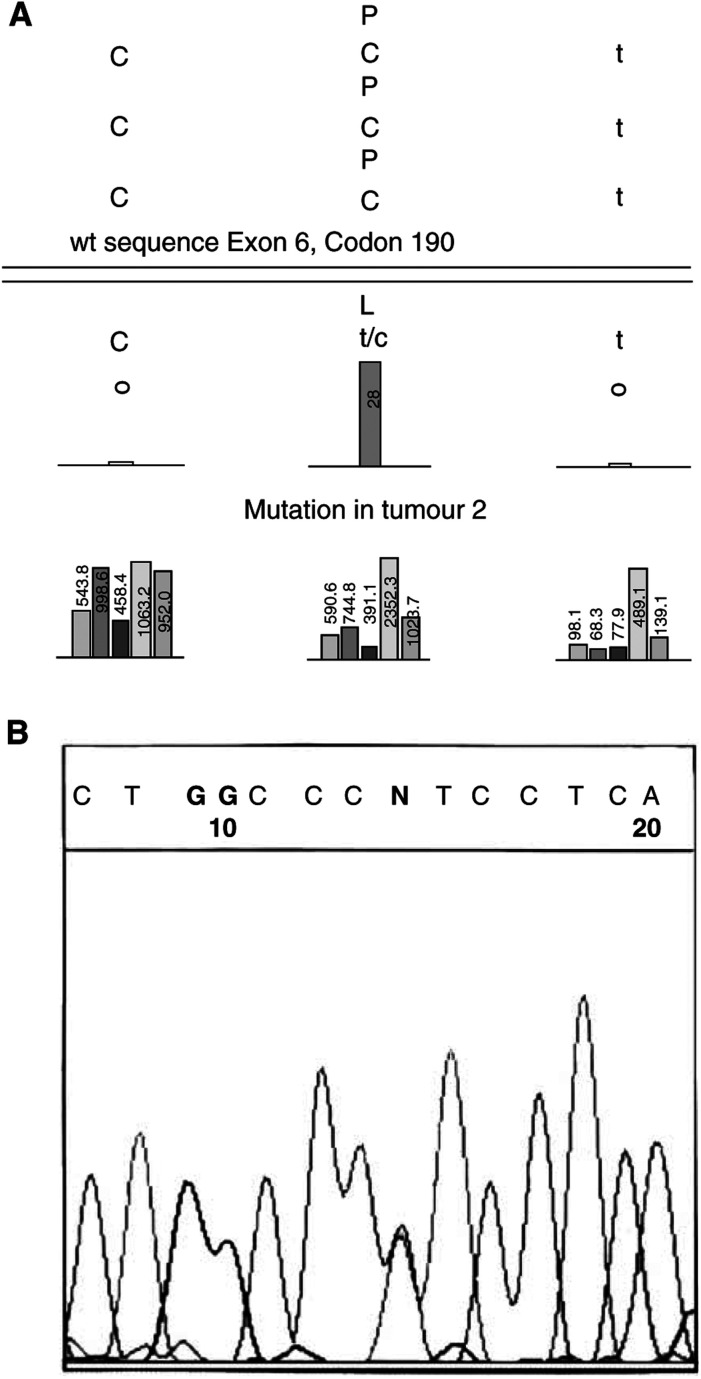
TP53 analysis of tumour 2 by P53GeneChip™ and by Sanger dideoxy sequencing. (**A**) Bar chart shows signal intensities from array tiling that screened codon 190 from a P53GeneChip hybridised with target from tumour 2. The C to T transition at codon 190 is indicated by the bar at position 2 [C **C** T to C **T**/**C** T, Proline (P) to Leucine (L)]. (**B**) Electropherogram from ABI Genetic Analyzer showing p53 exon 6 DNA sequence (5′ to 3′) in tumour 2. The ‘N’ shows the position of the C to T transition at the second position of codon 190.

We did not find mutations in exon 2 of the KLF6 gene in any of the 32 tumour samples that we examined (Table 1) nor in the human prostate tumour cell lines PC-3, DU-145 and LNCaP. The P53GeneChip™ protocol identified TP53 gene mutations in three of the 25 tumours (12%). All were transitions at G : C base pairs (the most common type of mutation in this cancer type) at codons previously reported mutated in prostate and other cancers (Olivier *et al*, 2002). The presence of these mutations was confirmed by amplifying genomic DNA with p53-specific primers, and then performing cycle sequencing and electrophoretic analysis following the same standard methodology used for KLF6 analysis (Table 1, Figure 1

).

Results of the TP53 mutation analysis were as expected from data in the scientific literature; however, the absence of KLF6 miscoding mutations in our patients and in the three human prostate cancer cell lines was unexpected. If KLF6 mutations were a common event in primary prostate cancer, it is likely that most tumour cell lines would harbour KLF6 mutations, because tumour cell lines typically harbour specific oncogene or tumour suppressor gene mutations at still higher prevalence than the frequencies detected in the corresponding primary tumours, and analysis is not plagued by particular technical difficulties such as contamination of tumour cells with non-neoplastic tissue.

Our findings on 32 European patients with prostate cancer (average Gleason score 7.3) suggest that KLF6 miscoding mutations in tumours of the prostate are uncommon. While the present report was under initial review, a new study on KLF6 alterations in prostate tumours appeared in the *American Journal of Pathology* reporting a low prevalence of miscoding KLF6 mutations in tumours of patients with high-grade prostate cancer (9%, average Gleason score >8, 75 tumours analysed; Chen *et al*, 2003). None of the mutations found by Chen *et al* occurred in the zinc-finger domains of KLF6, and none was identical to the mutations discovered by Narla *et al*. Thus, both the findings of Chen *et al* and our results contrast with the data in the first study (Narla *et al*, 2001), which suggested that KLF6 mutations are a common feature of prostate cancer (55%, 19 out of 34 tumour samples, average Gleason score 6.3). On the basis of information in these three studies, it is not likely that the discrepancies are attributable to differences in tumour grade of sample sets, whereas patient ethnicity (not given in the publications cited) and details of mutation analysis protocols merit attention in further investigations on the role of KLF6 mutations in prostate cancer.

Additional studies are called for in order to clarify whether KLF6 is indeed a common mutational target in prostate carcinogenesis.
